# Whole-Genome Sequences of 80 Environmental and Clinical Isolates of Burkholderia pseudomallei

**DOI:** 10.1128/genomeA.01282-14

**Published:** 2015-02-12

**Authors:** Shannon L. Johnson, Anthony L. Baker, Patrick S. Chain, Bart J. Currie, Hajnalka E. Daligault, Karen W. Davenport, Christopher B. Davis, Timothy J. J. Inglis, Mirjam Kaestli, Sergey Koren, Mark Mayo, Adam J. Merritt, Erin P. Price, Derek S. Sarovich, Jeffrey Warner, M. J. Rosovitz

**Affiliations:** a Bioenergy and Biome Sciences Group, Bioscience Division, Los Alamos National Laboratory, Los Alamos, New Mexico, USA; b College of Public Health, Medical and Veterinary, James Cook University, Townsville, Queensland, Australia; c Global and Tropical Health Division, Menzies School of Health Research, Casuarina, Northern Territory, Australia; d Institute for Glycomics, Griffith University, Queensland, Australia; e Division of Microbiology and Infectious Diseases, PathWest Laboratory Medicine Western Australia, Nedlands, Western Australia, Australia; f School of Pathology and Laboratory Medicine, The University of Western Australia, Nedlands, Western Australia, Australia; g National Biodefense Analysis and Countermeasures Center, Frederick, Maryland, USA

## Abstract

Here, we present the draft genome sequences of 80 isolates of Burkholderia pseudomallei. The isolates represent clinical cases of melioidosis and environmental isolates from regions in Australia and Papua New Guinea where B. pseudomallei is endemic. The genomes provide further context for the diversity of the pathogen.

## GENOME ANNOUNCEMENT

Burkholderia pseudomallei is the causative agent of melioidosis and is endemic in parts of the tropical world, including northern Australia, Papua New Guinea, and Southeast Asia (1–3). Studies of pathogen phylogeny or diversity using whole-genome sequencing have been dominated by Asian strains, for which more genome sequences were available (4, 5). We report here the whole-genome sequences of 80 B. pseudomallei isolates from both Australian clinical cases and environmental sampling of geographically diverse regions in northern Australia and Papua New Guinea. The genomes will contribute to our understanding of the global diversity of B. pseudomallei.

High-quality, high-molecular-weight genomic DNA was sequenced using a combination of Illumina, 454, and PacBio technologies, depending on the isolate. For those with only Illumina short-insert data (100-bp reads, noted as “I” in Table 1) assemblies were generated with IDBA version 1.1.1 (6). For those that also included Roche 454 data (noted as “R”) or Illumina long-insert data (insert sizes 8 to 10 kb, noted as “L”), the libraries were assembled together in Newbler version 2.6 (Roche) and the consensus sequences computationally shredded into 2-kbp overlapping fake reads (shreds). The raw reads were also assembled in Velvet and those consensus sequences computationally shredded into 1.5-kbp overlapping shreds (7). Draft data from all platforms were assembled together with AllPaths (8), and if Pacific Biosciences data was available (noted in Table 1 as “P”) and at 100× coverage or greater, assembled using HGAP (9). Consensus sequences from all assemblers were computationally shredded and assembled with a subset of read pairs from the long-insert library using Phrap (10, 11). The resulting assemblies were manually and computationally improved using Consed (12) and in-house scripts.

**TABLE 1:** B. pseudomallei isolate and assembly characteristics

Strain name	Isolation source^*a*^	GenBank accession no.	Sequence data type(s)^*b*^
MSHR44	Clinical, Australia	JQIM00000000	I, R, P
MSHR62	Clinical, Australia	CP009235, CP009234	I, R, P
MSHR303	Clinical, Australia	JQDD00000000	I, R, P
MSHR332	Clinical, Australia	JQFM00000000	I, R
MSHR435	Clinical, Australia	JRFP00000000	I, R, P
MSHR449	Clinical, Australia	JQFO00000000	I, R
MSHR456	Clinical, Australia	JQFN00000000	I, R, P
MSHR465J	Clinical, Australia	JPZW00000000	I, R, P
MSHR543	Clinical, Australia	JPZX00000000	I, R, P
MSHR640	Clinical, Australia	JQFP00000000	I, R, P
MSHR684	Clinical, Australia	JQDC00000000	I, R, P
MSHR733	Clinical, Australia	JQEE00000000	I, R, P
MSHR983	Clinical, Australia	JQDI00000000	I, R
MSHR1000	Clinical, Australia	JQEF00000000	I, R, P
MSHR1029	Clinical, Australia	JQDB00000000	I, R, P
MSHR1153	Clinical, Australia	CP009271, CP009272	I, R, P
MSHR1357	Clinical, Australia	JQDA00000000	I, R, P
MSHR2138	Clinical, Australia	JRFM00000000	I, R, P
MSHR2243	Clinical, Australia	CP009270, CP009269	I, R, P
MSHR2451	Clinical, Australia	JQEG00000000	I, R, P
MSHR2990	Clinical, Australia	JQHV00000000	I, R, P
MSHR3016	Clinical, Australia	JQEH00000000	I, R
MSHR3335	Clinical, Australia	JRFL00000000	I, R
MSHR3458	Clinical, Australia	JQOB00000000	I, R
MSHR3709	Clinical, Australia	JRFK00000000	I, R, P
ABCPW 1	−15.3150140, 126.1896240	JQIJ00000000	I, L, P
ABCPW 30	−16.0136890, 128.0230740	JPVF00000000	I, L, P
ABCPW 91	−15.3150140, 126.1896240	JPUY00000000	I, L, P
ABCPW 107	−15.3150260, 126.1898070	JQDN00000000	I
ABCPW 111	−16.5141220, 126.3560540	JPWT00000000	I
A79A	−8.0692000, 142.8755583	CP009165, CP009164	I, L, P
A79C	−8.0692000, 142.8755583	JQHQ00000000	I
A79D	−8.0692000, 142.8755583	JQHR00000000	I
BDU 2	−10.1579389, 142.1616056	JPVG00000000	I, L, P
B03	−8.0333333, 142.9500000	CP009151, CP009150	I, L, P
K42	−8.0577000, 143.0036833	CP009162, CP009163	I, L, P
MSHR3951	−12.8916220, 131.6061200	JPVA00000000	I, R, P
MSHR3960	−12.8913950, 131.6064850	JPVJ00000000	I, R, P
MSHR3964	−12.8913950, 131.6064850	JPVD00000000	I, R, P
MSHR3965	−12.7900970, 132.1780710	CP009153, CP009152	I, R, P
MSHR3997	−12.6554170, 132.5470450	JQII00000000	P
MSHR4000	−12.6552010, 132.5470110	JPVL00000000	I, R, P
MSHR4003	−12.4078040, 132.9343310	JPUZ00000000	I, R, P
MSHR4009	−12.4079700, 132.9342690	JQIL00000000	I, R, P
MSHR4012	−12.4079700, 132.9342690	JPVH00000000	I, R, P
MSHR4018	−12.4079700, 132.9342690	JQIK00000000	I, R, P
MSHR4032	−12.4083230, 132.9533260	JPQL00000000	I, R, P
MSHR4299	−13.8181900, 131.8313620	JPVC00000000	I, L, P
MSHR4300	−13.8179390, 131.8316290	JPQI00000000	I, R, P
MSHR4303	−13.8257680, 131.8331820	JPVM00000000	I, L, P
MSHR4304	−13.8258120, 131.8330280	JPOA00000000	I, L, P
MSHR4308	−13.8258120, 131.8330280	JPVB00000000	I, L, P
MSHR4372	−14.5251380, 132.8651370	JPQJ00000000	I, L, P
MSHR4375	−14.5246650, 132.8646830	JPVI00000000	I, L, P
MSHR4377	−14.5202880, 132.8633330	JPQH00000000	I, L, P
MSHR4378	−14.4901000, 132.2500880	JQDP00000000	I
MSHR4462	−13.2399580, 131.1084030	JPQM00000000	I, L, P
MSHR4503	−14.1693460, 130.1228070	JPQN00000000	I, L, P
MSHR4868	−13.4320160, 132.2744090	JQGZ00000000	I
MSHR5492	−20.6658631, 135.6153707	JQDO00000000	I
MSHR5569	−12.0483860, 134.2244300	JQDL00000000	I
MSHR5596	−12.2827850, 134.0835920	JQDE00000000	I
MSHR5608	−12.2876070, 134.0838240	JPWQ00000000	I
MSHR5609	−12.3519550, 134.1108660	JQDJ00000000	I
MSHR5613	−20.6659906, 135.6148314	JQDK00000000	I
MSHR7334	−13.1708260, 130.6744830	JQDF00000000	I
MSHR7343	−13.1709770, 130.6739790	JQDM00000000	I
MSHR7498	−14.1288333, 134.4440333	JQDH00000000	I
MSHR7500	−14.1420167, 134.4274833	JREN00000000	I
MSHR7504	−14.1103500, 134.4069500	JPWR00000000	I
MSHR7527	−14.1903333, 134.3715833	JPWS00000000	I
TSV5	−19.2573333, 146.7928056	JQGY00000000	I
TSV25	−19.2643611, 146.7998611	JPVK00000000	I, L, P
TSV28	−19.2630528, 146.7966556	JQHU00000000	I
TSV31	−19.2601667, 146.7941111	JPVE00000000	I, L, P
TSV32	−19.2546944, 146.8012222	JQHT00000000	I
TSV43	−19.2601667, 146.7941111	JPQK00000000	I, L, P
TSV44	−19.2630528, 146.7966556	JQGX00000000	I
TSV48	−19.2564694, 146.7898111	CP009161, CP009160	I, L, P
TSV202	−19.2806167, 147.0308833	CP009157, CP009156, CP009155, CP009154	I, L, P

a Isolation source is reported as clinical or as latitude and longitude for environmental isolates.

b Sequence data types are Illumina short-insert (I), Roche 454 (R), Illumina long-insert (L), and Pacific Biosciences (P).

For strains MSHR62 and MSHR3997, a 10-kb insert library was sequenced on the Pacific Biosciences platform. The assembly was generated by Celera Assembler version 8.0 (13) by previously described methods (14). The longest 25× of corrected sequences were assembled, and contigs composed of fewer than 10 sequences were omitted. Contigs were manually merged based on identified end overlaps to obtain the final assembly. The MSHR62 10-kb insert assembly was used to assist in gap closure and correction of the short-read assembly.

For all genomes, annotations were completed at the Los Alamos National Laboratory (LANL) using the Ergatis workflow manager (15) and in-house scripts. Of the 80 B. pseudomallei genomes assembled, nine are at finished quality (<1 error per 100,000 bp [16]), 49 are either noncontiguous finished or improved high-quality draft (IHQD) and available as scaffolded draft assemblies, and 22 assemblies are unscaffolded drafts.

### Nucleotide sequence accession numbers.

Genome accession numbers for the assemblies deposited in DDBJ/ENA/GenBank are listed in Table 1.
